# Burn Care Specialists’ Views Toward End-of-Life Decision-Making in Patients With Severe Burn Injury: Findings From an Online Survey in Australia and New Zealand

**DOI:** 10.1093/jbcr/irac030

**Published:** 2022-03-07

**Authors:** Lincoln M Tracy, Sandra Reeder, Michelle Gold, Heather J Cleland

**Affiliations:** School of Public Health and Preventive Medicine, Monash University, Melbourne, VIC, Australia; School of Public Health and Preventive Medicine, Monash University, Melbourne, VIC, Australia; Central Clinical School, Monash University, Melbourne, VIC, Australia; Palliative Care Service, Alfred Health, Melbourne, VIC, Australia; Victorian Adult Burns Service, Alfred Hospital, Melbourne, VIC, Australia

## Abstract

Burn care clinicians are required to make critical decisions regarding the withholding and withdrawal of treatment in patients with severe and potentially non-survivable burn injuries. Little is known about how Australian and New Zealand burn care specialists approach decision-making for these patients. This study aimed to understand clinician beliefs, values, considerations, and difficulties regarding palliative and end-of-life (EoL) care discussions and decision-making following severe burn injury in Australian and New Zealand burn services. An online survey collected respondent and institutional demographic data as well as information about training and involvement in palliative care/EoL decision-making discussions from nurses, surgeons, and intensivists in Australian and New Zealand hospitals with specialist burn services. Twenty-nine burns nurses, 26 burns surgeons, and 15 intensivists completed the survey. Respondents were predominantly female (64%) and had a median of 15 years of experience in treating burn patients. All respondents received little training in EoL decision-making during their undergraduate education; intensivists reported receiving more on-the-job training. Specialist clinicians differed on who they felt should contribute to EoL discussions. Ninety percent of respondents reported injury severity as a key factor in their decision-making to withhold or withdraw treatment, but less than half reported considering age in their decision-making. Approximately two-thirds indicated a high probability of death or a poor predicted quality of life influenced their decision-making. The three cohorts of clinicians had similar views toward certain aspects of EoL decision-making. Qualitative research could provide detailed insights into the varying perspectives held by clinicians.

The majority of burn-related fatalities occur in low-income countries.^1–4^ When faced with a patient with severe and potentially non-survivable injuries, specialist burn care clinicians are required to make critical treatment decisions in the immediate post-injury phase and beyond. In these situations, clinicians are supported by extensive knowledge of predictors of mortality and prognostic scoring systems (eg, Baux score).^5–10^ Additionally, a recent scoping review concluded that aspects of palliative care (used interchangeably with end-of-life [EoL] care) can be applied in a burn-specific context.^11^ This is somewhat counterintuitive, as patients with traumatic injuries often have unique needs compared with patients normally seen by specialist palliative care services (eg, end-stage cancer). Most burn deaths occur rapidly—within days^12^—meaning there are greater opportunities for palliative care involvement in the smaller proportion of patients whose injuries are considered non-survivable but do not succumb within 24 to 48 hours.

Few studies have attempted to understand clinicians’ thought processes in EoL decision-making by exploring their perceptions. The first study in this space was conducted by Metaxa and Lavrentieva in 2015, who surveyed 41 burn intensive care unit (ICU) physicians from America and Europe and identified a moral difference between withholding and withdrawing treatment.^13^*Withholding* treatment has been defined as “the decision to not commence life-sustaining treatment,” whereas *withdrawing* treatment as “the deliberate cessation of a life-sustaining treatment, without providing another one, in the awareness that it will lead to the patient’s death.” ^13, 14^ More recently, Cunningham et al surveyed 289 U.S.-based palliative care specialists and burns surgeons regarding the importance of setting care-related goals in burned geriatric patients.^15^ Both groups indicated that their specialty was better at conducting such discussions than the other, resulting in differing views regarding the optimum leadership style for this process. There has yet to be a study exploring clinician beliefs and considerations toward palliative and EoL decision-making discussions undertaken in the Australasian region.

The aim of this study was to explore beliefs, values, and considerations regarding palliative and EoL care discussions and decision-making following severe burn injury in Australian and New Zealand burn services. To achieve the study aims, we surveyed burns nurses, burns surgeons, and intensivists from Australian and New Zealand specialist burn services who are involved in making such decisions for burns patients.

## METHODS

### Design

An online survey reported according to the Checklist for Reporting Results of Internet E-Surveys (CHERRIES; Supplementary File 1).^16^

### Survey Development

The survey comprised two parts (Supplementary File 2). The first collected demographic and institutional data, while the second focused on training and involvement in EoL decision-making and discussions (eg, who is and should be involved in such discussions). The survey was based on previous work and themes identified from other literature.^11, 13, 15, 17^ Various modifications were incorporated to better align survey contents with our aims. Useability and technical functionality were tested prior to release.

### Ethics Approval and Consent Process

The Monash University Ethics Committee approved this study (project ID 28660). Details regarding survey length; where, how, and for how long data would be stored; the investigators; and the purpose of the study were presented on the landing page (Supplementary File 2). Completion of the survey indicated the participant had consented to the study. Participation was voluntary and anonymous. Respondents who were willing to participate in additional semi-structured interviews provided contact details via a separate form. These data were stored in a separate location and could not be linked.

### Recruitment

The Australian and New Zealand Burn Association (ANZBA; the peak body for health professionals who care for burn-injured patients in these countries) distributed the survey to all members via email. Members could share the public link with nonmembers. Emails inviting the membership to participate are included as Supplementary File 3. Survey data were collected between May and July 2021. At the time of initial survey distribution, ANZBA had 258 registered members, including 87 medical staff (58 surgeons, 6 anesthetists, and 1 intensivist, with the remaining members “doctors”) and 100 nursing staff. Reminder invitations were circulated at approximately 2, 4, and 8 weeks after the initial invitations. Participants were not compensated.

### Data Collection

Study data were collected using research electronic data capture (REDCap) tools hosted at Monash University. REDCap is a secure, web-based platform designed to support data capture for research studies.^18, 19^ Survey items were presented in a set (non-randomized) order and utilized adaptive questioning to ensure a streamlined experience for respondents who had not been involved in EoL decision-making processes. Survey screens/pages were numbered. Respondents were able to review and change responses prior to submission.

### Statistical Analysis

Descriptive statistics described participant demographics and summarized responses to each of the survey questions. Frequencies and percentages were used for categorical variables; mean and standard deviation (SD) or median and interquartile range (IQR) were used for continuous variables depending on their distribution. Responses between clinician groups were compared using chi-square analyses, one-way analysis of variance tests, and Kruskal-Wallis tests; *P*-values < .05 were considered significant. Bonferroni adjustments were used to correct for multiple comparisons. Data management and statistical analyses were performed using Stata Version 14 (StataCorp, TX, USA). Figures were produced in the R statistical environment version 4.0.3^20^ using the *tidyverse*^21^ and *RColorBrewer*^22^ packages.

## RESULTS

### Characteristics of Survey Sample

The survey was open from May 28, 2021, to July 29, 2021. Responses were received from 95 respondents. Seven respondents did not belong to the specialties of interest and were excluded. The response rate could not be calculated as the survey denominator was unknown. Seventy of the remaining 88 responses were complete (79.5% completion rate), comprising 29 nurses, 26 surgeons, and 15 intensivists (Table 1). Respondents had a median (IQR) age of 47 (40–54) years. Most of the respondents worked at services treating only adult patients.

**Table 1.** Survey respondent characteristics

	All Respondents (n = 70)	Burns Nurse (n = 29)	Burns Surgeon (n = 26)	Intensivist (n = 15)	*P*
Age, median (IQR) years^†^	47.0 (40.0, 54.0)	46.0 (38.0, 54.0)	50.0 (45.0, 63.0)	43.0 (39.0, 49.0)	.057
Female	44 (63.8%)	27 (93.1%)	10 (40.0%)	7 (46.7%)	<.001
Clinical experience, median (IQR) years^‡^	20.0 (9.3, 30.0)	24.0 (16.5, 32.5)	20.0 (9.3, 35.0)	10.0 (5.0, 15.0)	.007
Burns-specific experience, median (IQR) years	15.0 (8.0, 22.0)	20.0 (14.0, 28.0)	15.0 (9.0, 21.0)	10.0 (8.0, 13.0)	.022
Service treats					.065
Adult patients	44 (62.9%)	20 (69.0%)	12 (46.2%)	12 (80.0%)	
Pediatrics only or both	26 (37.1%)	9 (31.0%)	14 (53.8%)	3 (20.0%)	
Cared for patient who has experienced EoL process	68 (97.1%)	27 (93.1%)	26 (100.0%)	15 (100.0%)	.23
Discussed EoL with patient^§^	48 (70.6%)	11 (40.7%)	22 (84.6%)	15 (100.0%)	<.001
Participated in EoL meeting with HCP and family^||^	57 (83.8%)	18 (66.7%)	24 (92.3%)	15 (100.0%)	.006

*EoL*, end of life; *HCP*, healthcare professional; *IQR*, interquartile range.

Data are presented as frequency (percentage) unless otherwise specified. Reported *P* values relate to chi-square or Kruskal-Wallis tests that compared differences between nurse, surgeon, and intensivist responses. Seven respondents (two occupational therapists, one anesthetist, one health psychologist, one physiotherapist, one specialist pain medicine physician, and one otherwise undefined allied health specialist) did not belong to the specialties of interest and were excluded from analyses.

Data missing for †4 respondents, ‡1 respondent, ^§^2 respondents, and ||2 respondents.

Approximately two-thirds of respondents were female; a greater proportion of nurses were female compared with surgeons and intensivists. Nurses and surgeons had worked in a clinical environment for longer than intensivists. Nurses also had more burn-specific experience than intensivists. Almost all respondents had cared for a patient who had died. A greater proportion of surgeons and intensivists had discussed EoL care with a patient, their family, and other healthcare professionals compared with nurses. Compared with nurses and surgeons, intensivists reported receiving more training in EoL decision-making at all career stages apart from their undergraduate degree (Supplementary Table 1). Forty-five percent of respondents indicated that their service/hospital had a dedicated care protocol/pathway for EoL decision-making following burn injury; responses did not differ by specialist role (Supplementary Table 2). Respondents indicated that the medical/treating team were the most common party to document the decision to withhold or withdraw treatment (Supplementary Table 3).

### Involvement in Palliative Care/EoL Scenarios

Almost all respondents thought burns clinicians/surgeons and the patient (if able to) should attend EoL decision-making discussions, while 90% of respondents indicated family, the medical treatment decision-maker, or other support person should attend (Table 2). Nearly all burns nurses felt they should be involved in these discussions; a smaller proportion of burns surgeons and intensivists concurred. A greater proportion of intensivists thought ICU nurses should be involved in these discussions compared with burns nurses and surgeons. A smaller proportion of intensivists thought palliative care physicians should be involved in these discussions compared with burns nurses and surgeons. Only a small proportion of respondents thought hospital ethical or legal services should be involved in EoL decision-making discussion. The “other person” free-text responses indicated cultural support workers and psychologists/psychiatrists should also contribute to these discussions.

**Table 2.** Responses of who should attend end-of-life decision-making discussions

	Burns Nurse (n = 29)	Burns Surgeon (n = 26)	Intensivist (n = 15)	*P*
Patient (if able to)	29 (100.0%)	25 (96.2%)	15 (100.0%)	.42
Medical treatment decision maker	25 (86.2%)	24 (92.3%)	14 (93.3%)	.67
Family or other support person	28 (96.6%)	23 (88.5%)	12 (80.0%)	.21
Burns clinician or surgeon	29 (100.0%)	25 (96.2%)	15 (100.0%)	.42
Burns nurse	27 (93.1%)	15 (57.7%)	9 (60.0%)	.006
Hospital ethical services	10 (34.5%)	5 (19.2%)	<5	.098
Hospital legal services	<5	<5	<5	.78
ICU physicians	26 (89.7%)	25 (96.2%)	15 (100.0%)	.33
ICU nurses	17 (58.6%)	11 (42.3%)	14 (93.3%)	.006
ED physicians	17 (58.6%)	11 (42.3%)	7 (46.7%)	.46
ED nurses	12 (41.4%)	5 (19.2%)	<5	.19
Palliative care clinicians	20 (69.0%)	15 (57.7%)	<5	.008
Social workers	23 (79.3%)	15 (57.7%)	8 (53.3%)	.13
Religious advisor	18 (62.1%)	10 (38.5%)	6 (40.0%)	.16
Other person	<5	<5	<5	.36

*ED*, emergency department; *ICU*, intensive care unit.

Respondents could select as many responses as they wanted, acknowledging that all possible attendees may not apply to every patient.

The greatest proportion of respondents reported that burn clinicians/surgeons attended EoL decision-making discussions, followed by ICU physicians, the patient, and their family or other support person (Table 3). A greater proportion of burns surgeons reported family or other support personnel being involved in these discussions compared with burns nurses and intensivists, but a greater proportion of intensivists reported the involvement of ICU nurses. Compared with intensivists, a greater proportion of burns nurses and surgeons reported that palliative care physicians attended these discussions. Cultural support workers and psychologists, psychiatrists, and mental health services were again reported as “other” personnel involved in these discussions.

**Table 3.** Responses of who is involved in end-of-life decision-making discussions

	Burns Nurse (n = 29)	Burns Surgeon (n = 26)	Intensivist (n = 15)	*P*
Patient (if able to)	22 (75.9%)	23 (88.5%)	12 (80.0%)	.48
Medical treatment decision maker	19 (65.5%)	20 (76.9%)	11 (73.3%)	.64
Family or other support person	22 (75.9%)	25 (96.2%)	10 (66.7%)	.039
Burns clinician or surgeon	27 (93.1%)	26 (100.0%)	13 (86.7%)	.20
Burns nurse	12 (41.4%)	11 (42.3%)	6 (40.0%)	.99
Hospital ethical services	<5	<5	0 (0.0%)	.56
Hospital legal services	<5	<5	0 (0.0%)	.75
ICU physicians	24 (82.8%)	22 (84.6%)	15 (100.0%)	.24
ICU nurses	6 (20.7%)	5 (19.2%)	10 (66.7%)	.002
ED physicians	15 (51.7%)	8 (30.8%)	<5	.16
ED nurses	<5	<5	<5	.25
Palliative care clinicians	9 (31.0%)	10 (38.5%)	0 (0.0%)	.024
Social workers	10 (34.5%)	12 (46.2%)	6 (40.0%)	.68
Religious advisor	<5	6 (23.1%)	<5	.41
Other person	<5	<5	<5	.99

*ED*, emergency department; *ICU*, intensive care unit.

Respondents could select as many responses as they wanted, acknowledging that all possible attendees may not apply to every patient.

Four out of five respondents felt burns clinicians/surgeons should lead EoL decision-making discussions, while approximately two-thirds of respondents felt ICU physicians should lead (Table 4). A greater proportion of burns nurses and surgeons felt a burn clinician should lead the discussions. A greater proportion of intensivists felt an ICU physician should lead the discussion. Compared with intensivists, a greater proportion of burns nurses and surgeons reported that palliative care physicians lead these discussions. Respondents who indicated “other personnel” should lead EoL decision-making discussions commented that anyone involved in the care of the patient should be able to *initiate* such discussions.

**Table 4.** Responses of who should lead end-of-life decision-making discussions

	Burns Nurse (n = 29)	Burns Surgeon (n = 26)	Intensivist (n = 15)	*P*
Patient (if able to)	6 (20.7%)	8 (30.8%)	<5	.41
Medical treatment decision maker	8 (27.6%)	7 (26.9%)	<5	.85
Family or other support person	5 (17.2%)	5 (19.2%)	<5	.54
Burns clinician or surgeon	24 (82.8%)	24 (92.3%)	8 (53.3%)	.010
Burns nurse	<5	<5	0 (0.0%)	.56
Hospital ethical services	<5	0 (0.0%)	0 (0.0%)	.23
Hospital legal services	<5	<5	0 (0.0%)	.75
ICU physicians	16 (55.2%)	14 (53.8%)	14 (93.3%)	.022
ICU nurses	<5	0 (0.0%)	0 (0.0%)	.49
ED physicians	8 (27.6%)	<5	<5	.027
ED nurses	<5	0 (0.0%)	0 (0.0%)	.49
Palliative care clinicians	9 (31.0%)	<5	0 (0.0%)	.037
Social workers	<5	<5	0 (0.0%)	.45
Religious advisor	<5	<5	0 (0.0%)	.56
Other person	<5	<5	0 (0.0%)	.75

*ED*, emergency department; *ICU*, intensive care unit.

Respondents could select multiple responses.

**Figure 1.**
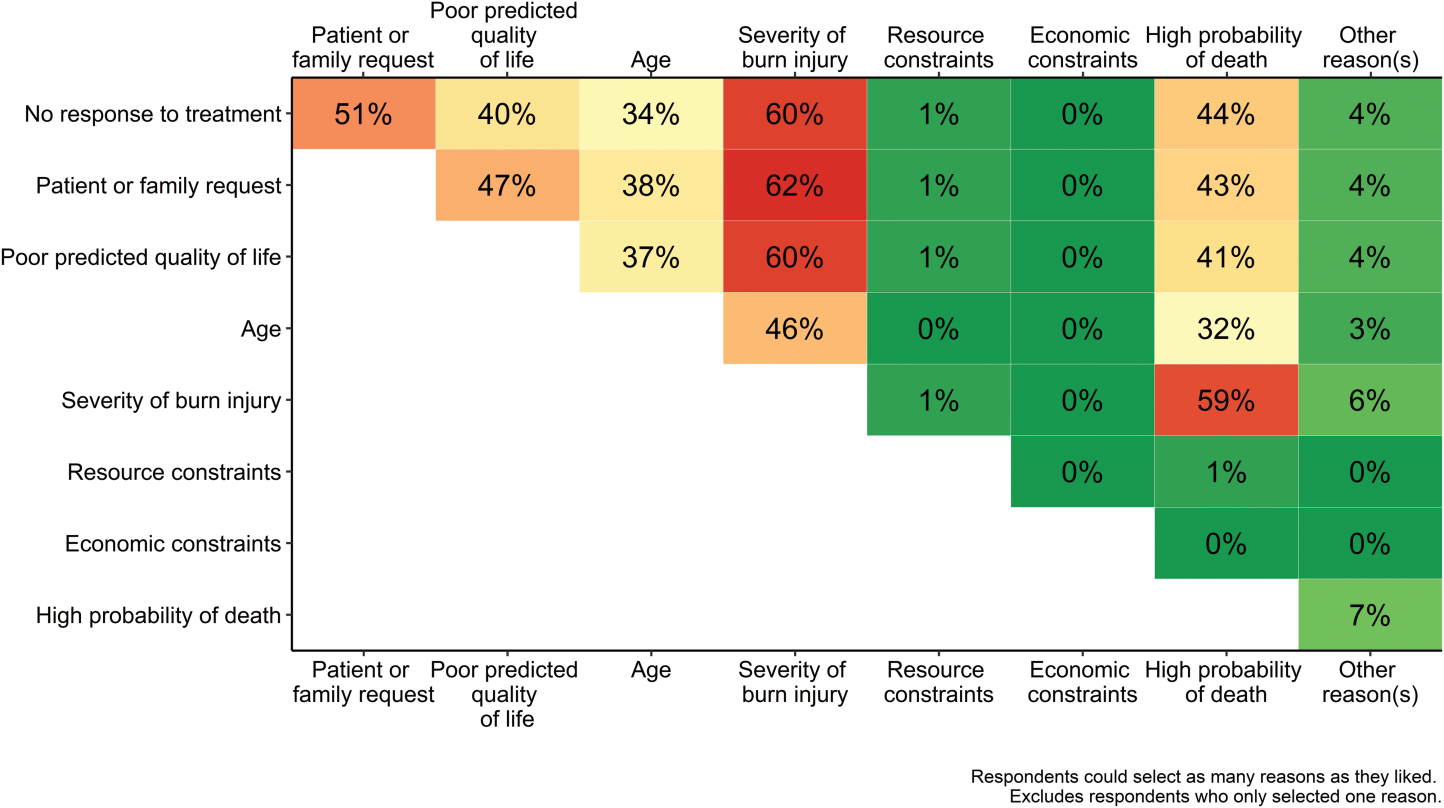
Proportion of respondents who select pairs of reasons when considering to withhold or withdraw treatment. Data presented as the proportion of respondents who selected multiple reasons. Excludes respondents who only selected one reason. Green represents fewer respondents selecting that pair of reasons, red represents more respondents selecting that pair of reasons (See Online version for color figure).

When asked about the most common reasons they would consider withholding or withdrawing treatment for a patient with a non-survivable burn injury, the greatest proportion of respondents selected injury severity (Table 5). Less than half of respondents indicated that they considered patient age when deciding to withdraw or withhold treatment. A greater proportion of intensivists indicated they consider a poor predicted quality of life compared with burns nurses or surgeons. A greater proportion of both intensivists and burns surgeons considered a high probability of death compared with burns nurses. Economic or resource constraints were rarely factored into decision-making. Other reasons for withholding or withdrawing treatment not listed in the survey (but listed by respondents in the accompanying “Other” free-text field) included significant or complex preexisting comorbidities or diagnoses (eg, terminal cancer diagnosis and psychological issues leading to repeated suicide attempts) and complications developing or worsening during the active treatment period. The mean (SD) number of reasons selected by responses was 4 (1.5; range: 1–7). There was no significant difference between the number of responses selected by specialist group (*F*(2,67) = 2.28, *P* = .11; Supplementary Table 4). All bar two respondents considered at least two reasons when deciding to withdraw or withhold treatment. Sixty-two percent of respondents who selected multiple reasons considered the severity of the burn injury and requests from the patient or family (Figure 1). Differences in the patterns of commonly selected responses between specialist burn care cohorts were observed (Supplementary Figure 1).

**Table 5.** Common reasons considered when deciding to withhold or withdraw treatment from patient with non-survivable burn injury

	Burns Nurse (n = 29)	Burns Surgeon (n = 26)	Intensivist (n = 15)	*P*
No response to treatment	20 (69.0%)	15 (57.7%)	9 (60.0%)	.67
Patient or family request	18 (62.1%)	14 (53.8%)	12 (80.0%)	.25
Poor predicted quality of life	20 (69.0%)	11 (42.3%)	14 (93.3%)	.004
Age	12 (41.4%)	10 (38.5%)	10 (66.7%)	.18
Severity of burn injury	28 (96.6%)	23 (88.5%)	12 (80.0%)	.21
Resource constraints	<5	0 (0.0%)	0 (0.0%)	.49
Economic constraints	0 (0.0%)	0 (0.0%)	0 (0.0%)	—
High probability of death	14 (48.3%)	21 (80.8%)	11 (73.3%)	.03
Other reason(s)	<5	<5	<5	.52

*ED*, emergency department; *ICU*, intensive care unit.

Respondents could select multiple responses.

## Discussion

This study presents the first survey of Australian and New Zealand burn care specialists’ views toward and experience in EoL decision-making discussions for patients with severe burns. Ninety-nine percent of respondents felt patients should be involved in EoL discussions if they were able to, while 81% of respondents indicated that family members were involved in such discussions. Ninety percent of respondents reported injury severity as a key factor in their decision-making to withhold or withdraw treatment. Views and experiences differed between the three specialist groups.

We are only able to make limited comparisons regarding the demographic characteristics of survey respondents of the current study with those of previous studies due to differences in the specific cohorts that were recruited.^13, 15^ A consistent finding to previously published work was burn surgeons reporting receiving less relevant training compared with other cohorts.^15^ Another consistent finding was both burns surgeons and intensivists indicating they felt they should lead EoL decision-making discussions. This parallels the findings of Cunningham et al, where burns surgeons and palliative care physicians viewed themselves as more adequate at performing and leading goals of care discussions than the other specialty.^15^ However, respondents in both the current study and the Cunningham et al’s study viewed that discussions such as these should be made in a more collaborative environment rather than being dictated by a single individual. It is important to consider that intensivists may feel better suited to lead EoL decision-making discussions when the patient is in the ICU; intensivists may have less involvement in EoL discussions for patients who remain on the ward.

Data from the current study paint a more collaborative picture of these discussions compared with previous research; only 46% of intensivist respondents in the Metaxa and Lavrentieva’s study indicated that they would involve nursing colleagues, while only 27% would engage the referring team.^13^ The range of different specialties involved in the decision-making process may also reflect the individualized nature of said processes: different professional support or expertise may be required for different patients and in different contexts. Multidisciplinary team involvement is considered best practice when treating patients with significant burn injuries and has been shown to reduce the risk of in-hospital mortality,^23–25^ and the potential benefits of integrating multidisciplinary and palliative care in burn services have been discussed elsewhere.^26^ Therefore, more clinicians should consider the potential benefits of taking a more multidisciplinary approach to EoL discussions and decision-making. A greater proportion of respondents in the current study also indicated that families should be and are involved in the decision-making process compared with Metaxa and Lavrentieva’s study.^13^

Approximately half of the respondents indicated that their service had a dedicated protocol/pathway for EoL decision-making following burn injury. From one perspective, it is somewhat unexpected that such a highly personalized and context-specific process could be guided by a protocol. On the other hand, protocols and frameworks can be used to ensure all people who *should* be involved in important EoL discussions are contacted and consulted in an appropriate and timely manner. They can also be useful when skills or knowledge in an area is lacking or where a process does not occur frequently enough to become embedded in practice. In addition, protocols can help ensure consistency within and between teams.

The greatest proportion of respondents indicated that the severity of the injury came into consideration when deciding to withhold or withdraw treatment. This is consistent with the findings of Metaxa and Lavrentieva, where 78% of respondents selected this option.^13^ This is not a particularly unexpected finding, as injury severity (eg, burn size and the presence of an inhalation injury) is a key contributor to prognostic scoring systems.^7, 10^ One unexpected finding was the low proportion of respondents who consider patient age when deciding to withhold or withdraw treatment. Age may already be considered in more commonly selected reasons (eg, high probability of death and/or poorer predicted quality of life). On average, respondents indicated that they considered four of the listed reasons during their decision-making process, and the majority consider several listed factors. These findings reinforce the complex and multifaceted nature of such decision-making processes required for patients with severe and potentially non-survivable burn injuries.

There were further similarities with previous work in other more commonly selected reasons, including a lack of response to treatment and a high predicted probability of death. Two interesting inconsistencies appeared when comparing the current findings to those of Metaxa and Lavrentieva. First, approximately two-thirds of respondents in the current study indicated that they considered requests from the patient and/or family when deciding to withhold or withdraw treatment compared with one-third of respondents in the Metaxa and Lavrentieva’s study. Second, a greater proportion of respondents in the current study compared with the Metaxa and Lavrentieva’s study included a poor predicted or expected outcome in their decision-making (63% and 44%, respectively). Personal experiences or beliefs (eg, religious^13^ or cultural) and variations in hospital models of care may underlie these differences.

Several differences in EoL decision-making experiences and views were reported between specialist cohorts. Intensivists reported receiving a greater amount of relevant training and on-the-job experience (including being directly involved in EoL decision-making discussions with patients and families) compared with burns surgeons and nurses. Having adequate and appropriate training and experience in EoL decision-making discussions may influence the views of intensivists, such as how a smaller proportion of intensivists reported involving palliative care physicians when treating patients with non-survivable burn injuries. In contrast, the less experienced burns nurses and surgeons may engage palliative care physicians in these discussions more frequently. Alternatively, these differences may arise through the unique aspects or challenges of dealing with death on the ward compared with the ICU. Research involving ICU nurses has identified the need for specific palliative care training and education programs amongst this specialty^26^; similar initiatives may be of use to burns surgeons and nurses. Further research into how experience influences the decision-making process is required.

Our study design resulted in the recruitment of a broader range of burn care clinicians and a higher completion rate compared with previous survey studies undertaken in this area.^13, 15^ Multiple reminder emails (to prompt potential participants who may have missed/ignored the initial invitation) were circulated in the weeks after the initial invitation was distributed, whereas previous studies were one-shot email distribution campaigns.^13, 15^ These reminders played a key role in the number of responses received and the high completion rate. However, the limitations of this study should also be considered. First, this study only surveyed burns nurses, burns surgeons, and intensivists. Responses to several items indicated that EoL discussions and decision-making should and do involve a broader, more multidisciplinary team beyond these three. Consequently, the current results may not reflect the complete views and opinions of all clinicians involved in this decision-making across Australia and New Zealand. Second, the recruitment strategy utilized for this study meant we were unable to determine the study denominator and calculate the response rate. This limits our ability to compare with other studies reporting this metric.^13, 15^ Finally, our sample size was smaller than that of Cunningham et al (70 in the current study compared with 289 in the previous study).^15^ However, the groups utilized for recruitment in the previous study (the American Burn Association and the American Academy for Hospice and Palliative Medicine) have larger memberships than ANZBA (>2000^27^ and >5000,^28^ respectively). Therefore, the current study had a smaller pool of potential participants to recruit from.

## Conclusions

This is the first study to explore clinician beliefs, values, and considerations regarding EoL care decision-making processes of burn care specialists in an Australian and New Zealand setting. These results, which are overall consistent with those obtained in an international setting, still reveal interesting insights into the EoL decision-making process in Australian and New Zealand burn care specialists. Further qualitative research on this topic could provide detailed insights into decision-making experience and how/why particular attitudes/beliefs exist.

## Supplementary Material

irac030_suppl_Supplementary_File_S1

irac030_suppl_Supplementary_File_S2

irac030_suppl_Supplementary_File_S3

irac030_suppl_Supplementary_File_S4

## Notes

This research was supported by a grant from the Bethlehem Griffiths Research Foundation (grant number BGRF2018). The funding source played no role in study design; the collection, analysis, and interpretation of data; the writing of the manuscript; or the decision to submit the manuscript for publication.

Data Availability As per approval from the Monash University Human Research Ethics Committee, this dataset is subject to ethical restrictions. The data are not publicly available due to information contained that could compromise the privacy of research participants.
