# Supplementary material for: Burn Care Specialists’ Views Toward End-of-Life Decision-Making in Patients With Severe Burn Injury: Findings From an Online Survey in Australia and New Zealand
Source: J Burn Care Res. 2022 Mar 7;43(6):1322–8. doi: 10.1093/jbcr/irac030 (PMC9629441; doi:10.1093/jbcr/irac030)
Supplement: irac030_suppl_Supplementary_File_S2 [file irac030_suppl_supplementary_file_s2.pdf]

Clinician Attitudes Regarding Palliative Care Following Burn Injury

Explanatory Statement

Project Title: Clinician Attitudes Regarding Palliative Care Following Burn Injury

Project Number: [redacted]

Project Team:

[redacted]

Welcome and thank you for your interest in this survey. Our team of researchers at the [redacted] are undertaking new research into clinician attitudes regarding palliative care following burn injury.

We are inviting you to participate in this survey. This Explanatory Statement contains detailed information about the survey. Its purpose is to explain to you openly and clearly all the procedures involved in this project before you decide whether or not to take part. Completion of the online survey will be considered consent to participate.

Why are we doing the survey?

We are interested in finding out more about clinician attitudes towards and experiences in palliative care following burn injury. This survey takes about 10 minutes to complete. The information gained from this survey provide essential data for informing palliative care decision-making following burn injury in Australia and New Zealand. Furthermore, the lessons learned from this project may translate to models of care for other patient groups who historically have had limited access to palliative care.

What is involved?

This is a voluntary and anonymous online survey containing questions about your views and attitudes towards palliative care practices following non-survivable burn injuries. No personal information is collected or stored through the survey.

While we will not ask for your name, there are questions relating to your age, gender, and occupation. If you work in an uncommon occupation, data regarding your age and gender may make your response less anonymous. However, data will be presented only in group form, and your particular response cannot be tracked back to you. As your particular response cannot be tracked back to you, it is not possible to withdraw from the study after you submit your survey.

What are the potential risks of participating?

There are no foreseeable risks to participants.

How will my confidentialty be handled and where will the data be stored?

Your data will be anonymous. A designated project folder will be created on [redacted] secure S:drive and accessible only to approved study personnel. All project documentation and data will be stored in this folder. Data will be stored for seven years following the completion of the project. All data besides your email address (if entered) may be made accessible to other researchers undertaking ethics committee approved research projects at the discretion of this project's researchers.

How will I find out the results?

The results of the survey findings will be presented at academic conferences and in-peer reviewed journals. If you would like to be informed directly of the findings, please contact [redacted]

Ethical Guidelines

This project will be carried out according to the National Statement on Ethical Conduct in Human Research (March 2007) produced by the National Health and Medical Research Council of Australia. This statement has been developed to protect the interests of people who agree to participate in human research studies. The ethical aspects

of this research project have been approved by the [redacted] [redacted].

What do I do if I want to make a complaint?

Should you have any concerns or complaints about the conduct of the project, you are welcome to contact the Executive Officer, [redacted]

[redacted]

[redacted]

BLOCK ONE - DEMOGRAPHICS

Age (in years)	________________________________<br>(Please enter your age as a whole number (e.g., 35))
Gender	◯ Male<br>◯ Female<br>◯ Prefer not to say<br>◯ Other<br>(Please select the gender you identify with)
Other gender	________________________________<br>(Please describe the gender you identify with)
Role	◯ Surgeon<br>◯ Intensivist<br>◯ Nurse<br>◯ Other<br>(Please select the role that best describes you)
Other Role	________________________________
How long have you worked as a [role]?	________________________________<br>(Please enter your response in years (e.g., 4.25, 17, etc.))
How long have you worked as an [role]?	________________________________<br>(Please enter your response in years (e.g., 4.25, 17, etc.))
How long have you worked as a/an [role_other]?	________________________________<br>(Please enter your response in years (e.g., 4.25, 17, etc.))
How long have you worked in a burn service OR how long have you been providing specialised treatment to patients with burn injuries?	________________________________<br>(Please enter your response in years (e.g., 4.25, 17, etc.))

Does your burn service or hospital primarily treat:	◯ Adult patients<br>◯ Paediatric patients<br>◯ Both adult and paediatric patients<br>(Please indicate the type of service/hospital you work in)

BLOCK TWO - END-OF-LIFE (EoL) DECISION-MAKING

Definitions

Withholding treatment: the decision to not commence life-sustaining treatment

Withdrawing treatment: the deliberate cessation of a life-sustaining treatment, without providing another one, in the awareness that it will lead to the patient's death

How much training in end of life (EoL) decision-making did you receive during your undergraduate degree?

- ◯ None
- ◯
- ◯
- ◯ Some
- ◯
- ◯
- ◯ Extensive
- ◯ Not applicable

How much training in end of life (EoL) decision-making did you receive during your residency?

- ◯ None
- ◯
- ◯
- ◯ Some
- ◯
- ◯
- ◯ Extensive
- ◯ Not applicable

How much training in end of life (EoL) decision-making did you receive during your fellowship?

- ◯ None
- ◯
- ◯
- ◯ Some
- ◯
- ◯
- ◯ Extensive
- ◯ Not applicable

How much training in end of life (EoL) decision-making did you receive during your postgraduate course, training, or diploma?

- ◯ None
- ◯
- ◯
- ◯ Some
- ◯
- ◯
- ◯ Extensive
- ◯ Not applicable

How much training in end of life (EoL) decision-making have you received while working as a [role]?

- ◯ None
- ◯
- ◯
- ◯ Some
- ◯
- ◯
- ◯ Extensive
- ◯ Not applicable

Have you ever cared for a patient who has experienced an EoL decision-making/palliative care process with a non-survivable burn injury?

- ◯ No
- ◯ Yes
- ◯ Unsure

Have you ever discussed EoL care with a patient you were looking after?

- ◯ No
- ◯ Yes
- ◯ Unsure

Have you ever you been part of a meeting between clinicians and patients/their family where EoL decision-making was discussed?	◯ No<br>◯ Yes<br>◯ Unsure
If a decision to withdraw/withhold treatment is made for a patient with a non-survivable burn injury, who documents it in the history?	__________________________________
To the best of your knowledge, does your service/hospital have a dedicated care pathway/tool/protocol for EoL decision-making following a burn injury?	◯ No<br>◯ Yes<br>◯ Unsure
Who do you think should attend (or be involved in) meetings regarding EoL decision-making discussions for a non-survivable burn injury? Select all that apply, acknowledging that all possible attendees may not apply to every patient.	☐ Patient (if they have the capacity to)<br>☐ Medical treatment decision maker<br>☐ Family or other support person<br>☐ Burns clinician or surgeon<br>☐ Burns nurses<br>☐ Ethical services of the hospital<br>☐ Legal services of the hospital<br>☐ ICU physician<br>☐ ICU nurses<br>☐ ED physician<br>☐ ED nurses<br>☐ Palliative care clinicians<br>☐ Social workers<br>☐ Religious advisor<br>☐ Other
Other person/people who should attend (or be involved in) meetings regarding EoL decision-making:	__________________________________<br>(Please specify any other people/roles not listed above)
At your hospital, who is actually involved in EoL decision-making for patients with a non-survivable burn injury? Select all that apply, acknowledging that all possible attendees may not apply to every patient.	☐ Patient (if they have the capacity to)<br>☐ Medical treatment decision maker<br>☐ Family or other support person<br>☐ Burns clinician or surgeon<br>☐ Burns nurses<br>☐ Ethical services of the hospital<br>☐ Legal services of the hospital<br>☐ ICU physician<br>☐ ICU nurses<br>☐ ED physician<br>☐ ED nurses<br>☐ Palliative care clinicians<br>☐ Social workers<br>☐ Religious advisor<br>☐ Other<br>(Please select all that apply)
Other person/people who is/are involved in EoL decision-making at your hospital?	__________________________________<br>(Please specify any other people/roles not listed above)

Who of the following do you think should lead meetings regarding EoL decision-making with patients and/or their surrogates after admission with a non-survivable burn injury?	☐ Patient (if they have the capacity to)<br>☐ Medical treatment decision maker<br>☐ Family or other support person<br>☐ Burns clinician or surgeon<br>☐ Burns nurses<br>☐ Ethical services of the hospital<br>☐ Legal services of the hospital<br>☐ ICU physician<br>☐ ICU nurses<br>☐ ED physician<br>☐ ED nurses<br>☐ Palliative care clinicians<br>☐ Social workers<br>☐ Religious advisor<br>☐ Other<br>(Please select all that apply)
Other person/people who should lead EoL decision-making at your hospital?	__________________________________<br>(Please specify any other people/roles not listed above)
What are the most common reasons you would consider withdrawing or withholding treatment from a patient with a non-survivable burn injury? Please select all that apply.	☐ No response to treatment<br>☐ Patient or family request (including advance care directives)<br>☐ Predicted poor quality of life as an outcome<br>☐ Age<br>☐ Severity of burn injury<br>☐ Resource constraints or issues (e.g., bed required for a patient with a greater chance of survival)<br>☐ Economic constraints or issues<br>☐ High probability of death in the short-term<br>☐ Other reason(s)<br>(Please select all that apply)
Other reason(s) you would consider withdrawing or withholding treatment from a patient with a non-survivable burn injury?	____<br>(Please list any other reasons)

BLOCK THREE - END OF SURVEY

Before we finish the survey, we would like to let you know that there is an additional part to the study collecting additional information about clinician attitudes, considerations, and difficulties regarding palliative and end-of-life care following a non-survivable burn injury. This part of the study involves one telephone/video conferencing interview where you would have the opportunity to discuss your attitudes and experiences with palliative and end-of-life care in more detail. You do not have to take part if you don't want to. If you are interested in participating, a member of the project team will send you the participant information sheet for you to read in detail. After you have had the opportunity to read the study documentation, we will contact you again to see if you would like to take part and arrange a suitable time for the interview.

This concludes the survey. Please submit your responses.

Would you like to be sent the information?

- ◯ No
- ◯ Yes (please provide your email address and a project investigator will be in touch)

Please click this link to provide your details so the researchers can contact you to arrange an interview. Note that your details will not be linked to your responses to this survey and will not be used for any other purpose.
