# Supplementary material for: Burn Care Specialists’ Views Toward End-of-Life Decision-Making in Patients With Severe Burn Injury: Findings From an Online Survey in Australia and New Zealand
Source: J Burn Care Res. 2022 Mar 7;43(6):1322–8. doi: 10.1093/jbcr/irac030 (PMC9629441; doi:10.1093/jbcr/irac030)
Supplement: irac030_suppl_Supplementary_File_S3 [file irac030_suppl_supplementary_file_s3.pdf]

Initial Invitation

Subject:
Study Invitation: Clinician Attitudes Regarding Palliative Care Following Non-Survivable Burn Injury

My name is [redacted] and I am a researcher at [redacted]. I am conducting research in collaboration with [redacted].

We would like to invite you to take part in our online study. We are hoping to learn more about the attitudes, beliefs, and considerations regarding palliative and end-of-life care following non-survivable burn injury in Australian and New Zealand specialist burn services.

The information gained from this research will provide essential data for informing palliative care decision-making following burn injury in Australia and New Zealand. Furthermore, the lessons learned from this research may translate to models of care for other patient groups who historically have had limited access to palliative care.

We are inviting surgeons, intensivists, and nurses employed in Australian and New Zealand hospitals with specialists burn services to participate in our study.

If you would like to learn more about the study and read the full information sheet, please click the link below. This will redirect you to the study page hosted on REDCap. No information will be collected until you begin the survey.

Clinician Attitudes Regarding Palliative Care Following Burn Injury

If the link above does not work, try copying the link below into your web browser:
https://redcap.[redacted]edu/surveys/?s=HXPCJLYTYR

You are under no obligation to participate. If you do decide to take part, no personal information will be collected.

If you do not have the time to participate but would still like to help us out, please consider forwarding the link to your colleagues.

We will send a reminder email in one week, followed by two further reminders at 2- to 3-week intervals.

If you have any questions, please do not hesitate to contact me via email:
[redacted].

Thank you for considering taking part.

Kind regards,
[redacted]

First Reminder (Sent Two Weeks After Initial Invitation)

Subject:
First Reminder: Clinician Attitudes Regarding Palliative Care Following Non-Survivable Burn Injury

My name is [redacted] and I am a researcher at [redacted]. I am conducting research in collaboration with [redacted].

We would like to invite you to take part in our online study. We are hoping to learn more about the attitudes, beliefs, and considerations regarding palliative and end-of-life care following non-survivable burn injury in Australian and New Zealand specialist burn services.

The information gained from this research will provide essential data for informing palliative care decision-making following burn injury in Australia and New Zealand. Furthermore, the lessons learned from this research may translate to models of care for other patient groups who historically have had limited access to palliative care.

We are inviting surgeons, intensivists, and nurses employed in Australian and New Zealand hospitals with specialists burn services to participate in our study.

If you have already completed the survey, we thank you for your time. No further action is required.

If you would like to learn more about the study and read the full information sheet, please click the link below. This will redirect you to the study page hosted on REDCap. No information will be collected until you begin the survey.

Clinician Attitudes Regarding Palliative Care Following Burn Injury

If the link above does not work, try copying the link below into your web browser:
https://redcap.[redacted].edu/surveys/?s=HXPCJLYTYR

You are under no obligation to participate. If you do decide to take part, no personal information will be collected.

If you do not have the time to participate, or you have already participated and would still like to help us out, please consider forwarding the link to your colleagues.

We will send two further reminders at 2- to 3-week intervals.

If you have any questions, please do not hesitate to contact me via email:
[redacted].

Thank you for considering taking part.

Kind regards,
[redacted]

Second Reminder (Sent Four Weeks After Initial Invitation)

Subject:
Second Reminder: Clinician Attitudes Regarding Palliative Care Following Non-Survivable Burn Injury

My name is [redacted] and I am a researcher at [redacted]. I am conducting research in collaboration with [redacted].

We would like to invite you to take part in our online study. We are hoping to learn more about the attitudes, beliefs, and considerations regarding palliative and end-of-life care following non-survivable burn injury in Australian and New Zealand specialist burn services.

The information gained from this research will provide essential data for informing palliative care decision-making following burn injury in Australia and New Zealand. Furthermore, the lessons learned from this research may translate to models of care for other patient groups who historically have had limited access to palliative care.

We are inviting surgeons, intensivists, and nurses employed in Australian and New Zealand hospitals with specialists burn services to participate in our study.

If you have already completed the survey, we thank you for your time. No further action is required.

If you would like to learn more about the study and read the full information sheet, please click the link below. This will redirect you to the study page hosted on REDCap. No information will be collected until you begin the survey.

Clinician Attitudes Regarding Palliative Care Following Burn Injury

If the link above does not work, try copying the link below into your web browser:
https://redcap.[redacted].edu/surveys/?s=HXPCJLYTYR

You are under no obligation to participate. If you do decide to take part, no personal information will be collected.

If you do not have the time to participate, or you have already participated and would still like to help us out, please consider forwarding the link to your colleagues.

We will send one final reminder in approximately three weeks.

If you have any questions, please do not hesitate to contact me via email:
[redacted].

Thank you for considering taking part.

Kind regards,
[redacted]

Final Reminder (Sent Eight Week After Initial Invitation)

Subject:
Final Reminder: Clinician Attitudes Regarding Palliative Care Following Non-Survivable Burn Injury

My name is [redacted] and I am a researcher at [redacted]. I am conducting research in collaboration with [redacted].

We would like to invite you to take part in our online study. We are hoping to learn more about the attitudes, beliefs, and considerations regarding palliative and end-of-life care following non-survivable burn injury in Australian and New Zealand specialist burn services.

The information gained from this research will provide essential data for informing palliative care decision-making following burn injury in Australia and New Zealand. Furthermore, the lessons learned from this research may translate to models of care for other patient groups who historically have had limited access to palliative care.

We are inviting surgeons, intensivists, and nurses employed in Australian and New Zealand hospitals with specialists burn services to participate in our study.

If you have already completed the survey, we thank you for your time. No further action is required.

If you would like to learn more about the study and read the full information sheet, please click the link below. This will redirect you to the study page hosted on REDCap. No information will be collected until you begin the survey.

Clinician Attitudes Regarding Palliative Care Following Burn Injury

If the link above does not work, try copying the link below into your web browser:
https://redcap.[redacted].edu/surveys/?s=HXPCJLYTYR

You are under no obligation to participate. If you do decide to take part, no personal information will be collected.

If you do not have the time to participate, or you have already participated and would still like to help us out, please consider forwarding the link to your colleagues.

This is your final opportunity to complete the online survey. The survey will close in two weeks from the date of this email.

If you have any questions, please do not hesitate to contact me via email:
[redacted].

Thank you for considering taking part.

Kind regards,
[redacted]
