# Supplementary material for: Burn Care Specialists’ Views Toward End-of-Life Decision-Making in Patients With Severe Burn Injury: Findings From an Online Survey in Australia and New Zealand
Source: J Burn Care Res. 2022 Mar 7;43(6):1322–8. doi: 10.1093/jbcr/irac030 (PMC9629441; doi:10.1093/jbcr/irac030)
Supplement: irac030_suppl_Supplementary_File_S4 [file irac030_suppl_supplementary_file_s4.docx]

**Supplementary Materials**

| **Supplementary Table 1. End of life decision-making training received throughout career** | | | | |
| --- | --- | --- | --- | --- |
|  | **Burns Nurse**  **(n = 29)** | **Burns Surgeon**  **(n = 26)** | **Intensivist**  **(n = 15)** | **p-value** |
| Undergraduate degree^a^ | 1.0 (0.0, 2.5) | 2.0 (0.0, 3.0) | 1.0 (0.0, 2.0) | 0.29 |
| Residency^b^ | -- | 1.0 (0.0, 3.0) | 3.0 (2.0, 5.0) | 0.017 |
| Fellowship^c^ | -- | 2.0 (0.0, 3.0) | 5.0 (3.0, 6.0) | <0.001 |
| Postgraduate setting^d^ | 2.0 (2.0, 3.0) | 2.0 (0.0, 3.0) | 4.5 (3.5, 5.0) | 0.012 |
| On-the-job^e^ | 3.0 (1.5, 4.0) | 2.0 (0.0, 3.0) | 5.0 (4.0, 6.0) | <0.001 |
| Data presented as median (interquartile range) on a seven point scale with anchors of 0 (no experience), 3 (some experience), and 6 (extensive experience).  Reported *p* values relate to chi-square or Kruskal Wallis tests that compared differences between nurse, surgeon, and intensivist responses.  Data missing for ^a^10 respondents, ^b^5 respondents, ^c^6 respondents, ^d^18 respondents, and ^e^11 respondents. | | | | |

| **Supplementary Table 2. Dedicated protocol/pathway in place for end of life decision-making** | | | | |
| --- | --- | --- | --- | --- |
|  | **Burns Nurse**  **(n = 29)** | **Burns Surgeon**  **(n = 26)** | **Intensivist**  **(n = 15)** | **p-value** |
| Unsure/No | 11 (39.3%) | 16 (61.5%) | 11 (73.3%) | 0.07 |
| Yes | 17 (60.7%) | 10 (38.5%) | 4 (26.6%) |  |
| Data presented as frequency (percentage).  Data missing for one respondent. | | | | |

| **Supplementary Table 3. Party responsible for documentation of treatment decision** | | |
| --- | --- | --- |
| **Response** | **N** | **%** |
| Medical team | 22 | 24.7% |
| Intensivist | 18 | 20.2% |
| Consultant | 17 | 19.1% |
| Surgeon | 12 | 13.5% |
| Registrar | 12 | 13.5% |
| Doctor | 2 | 2.2% |
| Leader | 2 | 2.2% |
| Fellow | 1 | 1.1% |
| ED consultant | 1 | 1.1% |
| Resident | 1 | 1.1% |
| Nurse | 1 | 1.1% |
| Note: Respondents could enter more than one responsible party. | | |

| **Supplementary Table 4. Number reasons considered when deciding to withhold or withdraw treatment from patient with non-survivable burn injury** | | | |
| --- | --- | --- | --- |
| **Burns Nurse**  **(n = 29)** | **Burns Surgeon**  **(n = 26)** | **Intensivist**  **(n = 15)** | **p-value** |
| 4.0 (1.3) | 3.7 (1.6) | 4.7 (1.5) | 0.11 |
| Data presented as mean (standard deviation). | | | |


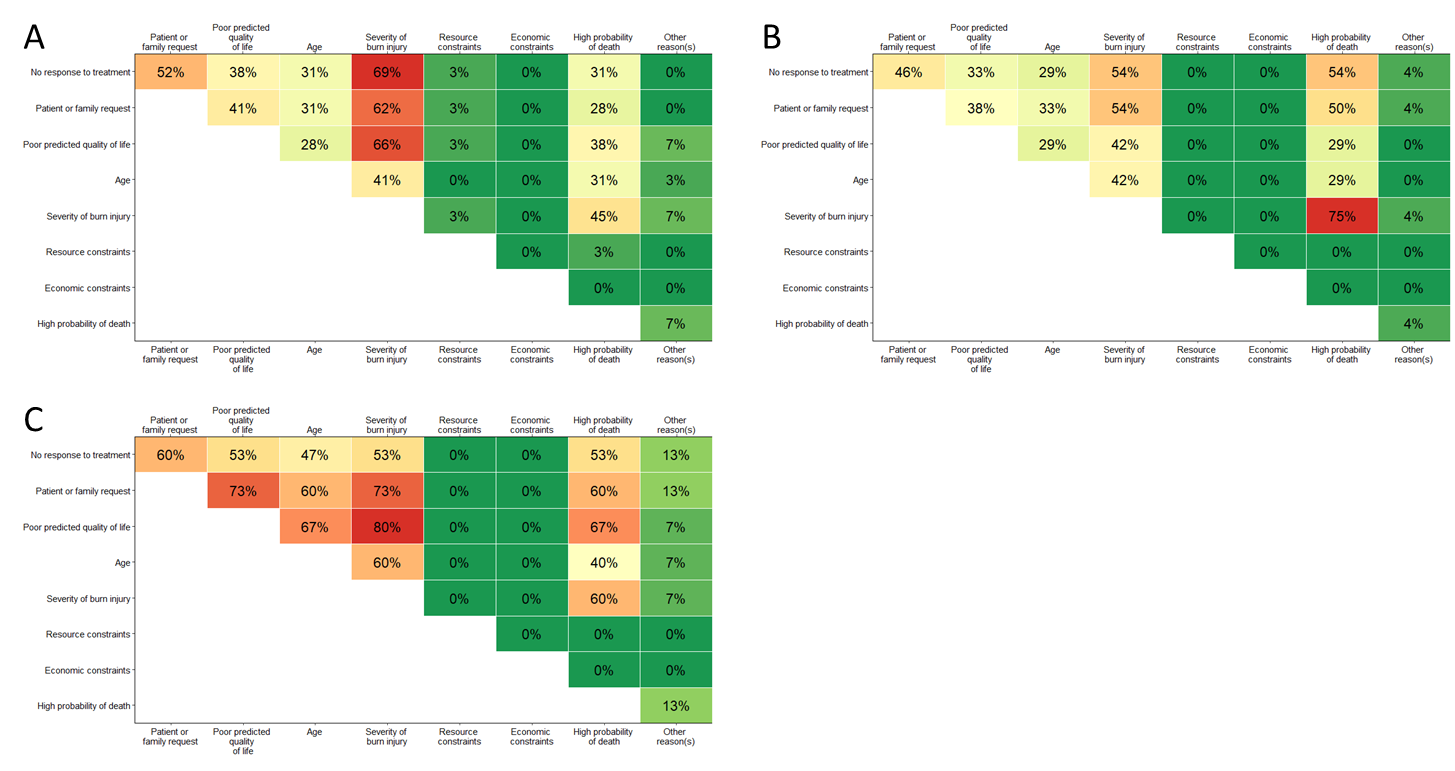


**Supplementary Figure 1.** Proportion of (a) burn nurse, (b) burn surgeon, and (c) intensivist respondents who select pairs of reasons when considering to withhold or withdraw treatment. Data presented as proportion of respondents who selected multiple reasons. Excludes respondents who only selected one reason.
